# Molecular Characterization of Chinese Hamster Cells Mutants Affected in Adenosine Kinase and Showing Novel Genetic and Biochemical Characteristics

**DOI:** 10.1186/1471-2091-12-22

**Published:** 2011-05-17

**Authors:** Xianying A Cui, Tanvi Agarwal, Bhag Singh, Radhey S Gupta

**Affiliations:** 1 Department of Biochemistry and Biomedical Sciences, McMaster University, Hamilton, L8N 3Z5, Canada

## Abstract

**Background:**

Two isoforms of the enzyme adenosine kinase (AdK), which differ at their N-terminal ends, are found in mammalian cells. However, there is no information available regarding the unique functional aspects or regulation of these isoforms.

**Results:**

We show that the two AdK isoforms differ only in their first exons and the promoter regions; hence they arise via differential splicing of their first exons with the other exons common to both isoforms. The expression of these isoforms also varied greatly in different rat tissues and cell lines with some tissues expressing both isoforms and others expressing only one of the isoforms. To gain insights into cellular functions of these isoforms, mutants resistant to toxic adenosine analogs formycin A and tubercidin were selected from Chinese hamster (CH) cell lines expressing either one or both isoforms. The AdK activity in most of these mutants was reduced to <5% of wild-type cells and they also showed large differences in the expression of the two isoforms. Thus, the genetic alterations in these mutants likely affected both regulatory and structural regions of AdK. We have characterized the molecular alterations in a number of these mutants. One of these mutants lacking AdK activity was affected in the conserved NxxE motif thereby providing evidence that this motif involved in the binding of Mg^2+ ^and phosphate ions is essential for AdK function. Another mutant, Fom^R^-4, exhibiting increased resistance to only C-adenosine analogs and whose resistance was expressed dominantly in cell-hybrids contained a single mutation leading to Ser_191_Phe alteration in AdK. We demonstrate that this mutation in AdK is sufficient to confer the novel genetic and biochemical characteristics of this mutant. The unusual genetic and biochemical characteristics of the Fom^R^-4 mutant suggest that AdK in this mutant might be complexed with the enzyme AMP-kinase. Several other AdK mutants were altered in surface residues that likely affect its binding to the adenosine analogs and its interaction with other cellular proteins.

**Conclusions:**

These AdK mutants provide important insights as well as novel tools for understanding the cellular functions of the two isoforms and their regulation in mammalian cells.

## Background

Adenosine kinase (AdK) is a major purine salvage pathway enzyme belonging to the ribokinase family of proteins [1-4]. It plays a central role in regulating the intracellular and interstitial concentrations of the purine nucleoside adenosine (Ado), which exhibits potent cardioprotective and neuroprotective activity [5-7]. During ischemia, the compromised regeneration of ATP causes an increase in the intracellular concentration of Ado, which results in its net efflux into extracellular space where it binds to G_i/o_-coupled Ado receptors: A_1_, A_2A_, A_2B_, and A_3_, to modulates a variety of physiological responses to reduce tissue damage from ischemic injury [5,6,8-10]. The expression of AdK undergoes rapid coordinated changes in the brain following epileptic seizures or stroke, resulting in an acute surge of Ado, which serves to minimize damage to the brain [6,11]. Strong evidence in support of the protective role of Ado has been obtained from studies where transient down regulation of AdK after acute brain injury protected brain from seizures and cell death, whereas its overexpression as in epilepsy caused seizure aggravation and promoted cell death [11-13].

AdK, in addition to its central role in purine salvage and ATP catabolism, also plays a critical role in the maintenance of methylation reactions. In the S-adenosylmethionine (SAM) dependent methylation pathway, Ado and homocysteine (Hcy) are produced as a result of hydrolysis of S-adenosyl-homocysteine (SAH), which is the common end product of all methylation reactions [1,14-17]. The hydrolysis reaction, which is catalyzed by the enzyme SAH-hydrolase, is reversible and the equilibrium constant of this reaction favors SAH formation. Hence, unless the hydrolysis product, Ado and Hcy are rapidly removed, it will lead to the buildup of SAH, which is a potent inhibitor of transmethylation reactions [14,17,18]. In the guinea-pig heart, the transmethylation pathway has been shown to be an important intracellular source of Ado under normal conditions and the Ado produced by this mechanism is mainly salvaged by AdK [19]. Studies with the AdK knockout mouse, which causes liver failure and early postnatal death [16], indicate that the effects of AdK deficiency on transmethylation reactions are the main underlying causes for its lethal effect [16]. The deficiency of AdK due to its pivotal role in the maintenance of transmethylation reaction also causes developmental abnormalities and reduced salt stress in plants [20,21].

Two isoforms of AdK are present in mammalian species [22-25]. These isoforms differ from each other only in their N-termini. The long isoform (AdK-L) of AdK contains an extra 20-21 amino acids in place of the first four amino acids of the AdK-short (AdK-S) isoform [23,26]. Studies with the recombinant AdK-L and AdK-S proteins have revealed no differences in their biochemical or kinetic properties [23](unpublished results). However, we recently showed that the N-terminal extension in the AdK-L functions as a nuclear localization signal [27]. Thus, of the two AdK isoforms, AdK-L is targeted to the nucleus whereas AdK-S is localized in the cytoplasm [27]. The differential subcellular localization of these two AdK isoforms suggests that they carry out different physiological functions. However, there is no information available at present regarding the unique cellular functions of these isoforms or how their expression is regulated.

Unlike the lethal phenotype of AdK^-/- ^mice, AdK deficient mutants can be readily obtained in cultured cells by selecting in the presence of toxic concentrations of the Ado analogs [25,28-31]. Most of such mutants lack AdK activity and some mutants that have been studied in detail contained large deletions within the AdK gene [24,32]. Our recent work shows that in contrast to the Chinese Hamster Ovary (CHO) cells that expresses only the AdK-L isoform, in the CH V79 cell line established from embryonic lung [33] both AdK-L and AdK-S isoforms are expressed. Hence, to gain further insights into the cellular functions of AdK, in the present work we report the isolation and characterization of mutants resistant to Ado analogs from V79 and other CH cell lines. Our results show that these mutants exhibit interesting differences in their cross-resistance pattern towards the N- and C- Ado analogs and also in the expression profiles of the two AdK isoforms. (Note: In N-nucleosides the purine base is linked to ribose via a N-C bond, whereas in C-nucleosides this linkage involves a C-C bond [34,35]). Several of these mutants contained novel molecular alterations in AdK affecting its activity/function. One of these mutants that exhibited increased resistance to only C-Ado analogs, and whose drug-resistance phenotype was dominantly expressed in cell hybrids formed with AdK^+ ^cells, has been characterized in detail [34]. We showed that a single point mutation in AdK is responsible for its novel genetic and biochemical characteristics. These mutants provide important insights and novel tools for understanding the cellular functions and regulation of the AdK isoforms in mammalian cells.

## Results

### The AdK-L and AdK-S Isoforms Differ in their First Exons and the Promoter Sequences

**Figure 1:**
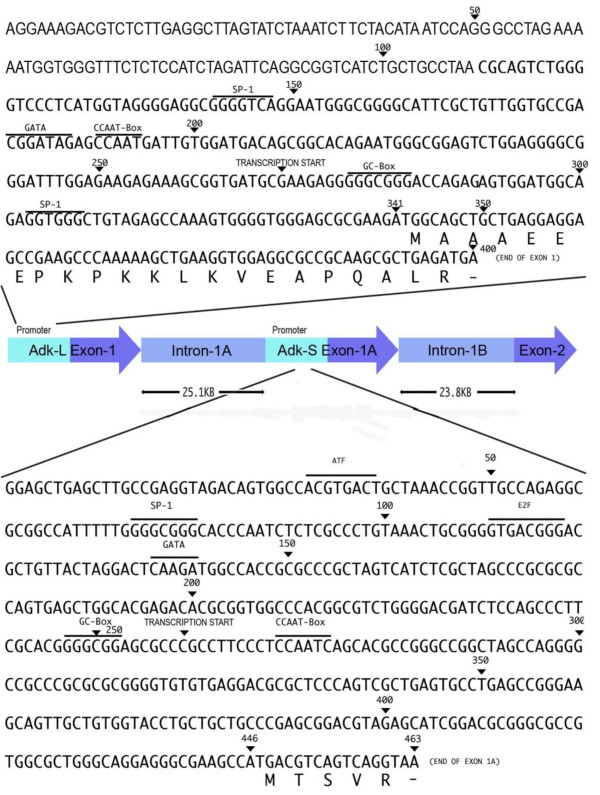
**A schematic drawing showing the genomic organization of the first exons and promoter regions for the AdK-L and AdK-S genes in the human genome**. The AdK gene (both AdK-L and AdK-S isoforms) in human is comprised of 11 exons. Except for the first exons, all the remaining exons (not shown here) are common to these isoforms. The Exon-1 and promoter region for the AdK-L isoform were identified in earlier work [32,36]. The intron-1 for the AdK-L is now shown to contain the first exon for the AdK-S isoform. The first exons for the AdK-L and AdK-S isoforms encode for all the amino acids that distinguish these two isoforms. The upstream regions of both exons contain binding sites for several transcriptional factors, only some of which are shown here. The introns and exons are not drawn to the scale in this diagram.

The AdK genes in human as well as rodent species are unusually large (human 546 kb and mouse 390 kb) and they consist of 11 exons that range in length from 36 to 765 nt [32,36]. We have previously shown that the promoter for the AdK-L isoform in human, CH and other mammals is bidirectional and it is linked in a head-to-head fashion with the gene encoding the clathrin adaptor mu3A protein [36]. The first exon of the AdK-L isoform contains the sequence information distinguishing it from the AdK-S isoform (Figure 1). The first exon and the promoter for the AdK-S isoform have not yet been identified. Our blast searches on the human genome with the nucleotide sequence specific for the AdK-S isoform and its upstream non-coding region [37] have revealed that it matches perfectly with a sequence within the first intron of the AdK-L isoform. This sequence region encodes for all amino acids that are distinctive of the AdK-S isoform (Figure 1, lower part). The analysis of the 3-4 kb region upstream of the AdK-S coding sequence using the EMBOSS program [38] indicates the presence of a promoter about 350 bp upstream of the initiator codon. This predicted promoter is located within a CpG island and several transcription factor binding sites are present in its proximity (Figure 1). Studies to determine the promoter activity of this region and the significance of various regulatory elements present in its vicinity will be undertaken in future. Because the AdK-L and AdK-S isoforms are identical except for the amino acids encoded by their first exons, the observed structural organization of the AdK gene indicates that the two AdK isoforms arise by differential splicing of their unique first exons with the other AdK exons. The fact that each of these exons has their own promoters strongly suggests that the expression of the two AdK isoforms is independently regulated at the transcriptional level.

### Expression of the Two Isoforms Differ in Various Tissues and Cell Lines

**Figure 2:**
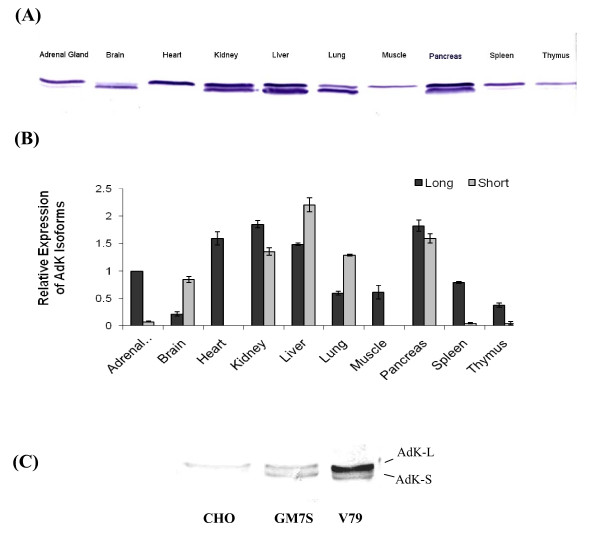
**Differences in expression of two AdK isoforms in various tissues and cell lines**. (A) Western blot showing the expression profile of the two isoforms in various rat tissues; (B) Quantification of the relative amounts of two AdK isoforms in rat tissues. Expression levels of the two isoforms were normalized relative to AdK-L level in adrenal gland and the average amount (intensity) ± SD for three independent experiments is shown. (C) Western blot showing relative expression of the two isoforms in CH CHO, V79 and GM7S cell lines.

The expression profile of AdK in rat tissues was examined by Western blotting (Figure 2A). In immunoblots of different tissue extracts, the antibody to AdK detected only two closely related protein species of molecular masses (Mr ≈ 40-42 KDa), which correspond to the two isoforms of AdK. In contrast to an earlier study, where three isoforms of AdK were reported [22], no third isoform was detected in our study. The expression of the two AdK isoforms varied markedly among different tissues. In liver, kidney, lung and pancreas both isoforms were expressed in comparable amounts (Figure 2A and 2B). In contrast, in heart, thymus and skeletal muscle, expression of mainly the AdK-L isoform was observed. Of the different tissues examined, brain was the only tissue where the short isoform was predominantly expressed (Figure 2A and 2B) [12]. The expression of the two AdK isoforms was also examined in a number of other mammalian cell lines. In contrast to the CHO lines that expressed mainly the AdK-L isoform, in the CH V79 and GM7 cell lines derived from embryonic lung [39], both AdK-L as well as AdK-S isoforms were expressed (Figure 2C). The expression of the two isoforms was comparable in the GM7S cells, whereas in V79 cell line the AdK-L isoform showed slightly higher expression. In our earlier work, the human Hela cells also showed expression of only the AdK-L isoform, whereas in human HT-1080 cell line and mouse LM (TK^-^) cells, both AdK isoforms were expressed in comparable amounts [27].

### Isolation and Characterization of Mutants Resistant to Adenosine Analogs from V79 Cells

To gain insights into the cellular functions of AdK and the regulation of its isoforms, mutants resistant to the Ado analogs tubercidin and formycin A (FoA) were selected from V79 cells. Of these analogs, tubercidin (and also toyocamycin) similar to adenosine is an N-nucleoside, whereas FoA is a C-nucleoside analog. In our earlier work, interesting differences have been observed in the cross-resistance patterns of AdK mutants towards the N- and C- Ado analogs [31,34]. Under the conditions employed (see Methods section), the resistant mutants were obtained at a frequency of ~ 2.5 × 10^-7^.

The degree of resistance of the mutants towards FoA and tubercidin was determined in comparison to the parental V79 cells. In the presence of FoA, the colony forming ability of WT V79 or CHO cells decreased sharply at drug concentrations between 5-10 ng/ml and no colonies were obtained at 20 ng/ml. For tubercidin, the colony forming ability of the WT cells decreased sharply between 2-5 ng/ml and no colonies were observed at 20 ng/ml. Based on these studies, the D_10 _values of the WT V79 cells for FoA and tubercidin were approximated to be 10 ng/ml and 5 ng/ml, respectively (Table 1). The degree of resistance of the mutant cell lines in comparison to the parental sensitive cell line was determined based on the ratios of their D_10 _values [40]. Based on their relative resistance to FoA and tubercidin, the different mutants that we have isolated could be divided into two groups (Table 1). All four Tub^R ^mutants (VT2 -5) and ten Fom^R ^mutants viz. VF2-8, VF20, VF24, and VF26, which exhibited >50-fold resistance to both FoA and tubercidin formed the first group. The resistance levels of these mutants to these analogs is similar to that for the toyocamycin and tubercidin resistant mutant of CHO cells (including the Toy^R^-4 and DrToy^R^-18) isolated previously [29,41]. In contrast to these mutants, ten other Fom^R ^mutants (VF1, VF9-13, VF15, VF18, VF19 and VF23) were between 10-50 fold resistant to FoA, but only showed minimal (2-5 fold) resistance to tubercidin. The drug-resistance profile of these mutants (Group B) is similar to that of the Fom^R^-4 mutant of CHO cell isolated in earlier work [34].

**Table 1:** Degree of Resistance of Mutants Cell Lines to Adenosine Analogs

V79 Cell Line	Relative Resistance of the Mutant Cell lines	
	
	Formycin A	Tubercidin
WT (A)	1 (~20 ng/ml)	1 (~10 ng/ml)

Tub^R^2 (A)	>50	>50

Tub^R^3 (A)	>50	>50

Tub^R^4 (A)	>50	>50

Tub^R^5 (A)	>50	>50

Fom^R^2 (A)	>50	>50

Fom^R^3 (A)	>50	>50

Fom^R^4 (A)	>50	>50

Fom^R^5 (A)	50	>50

Fom^R^6 (A)	>50	>50

Fom^R^7 (A)	>50	>50

Fom^R^8 (A)	>50	>50

Fom^R^20 (A)	>50	>50

Fom^R^24 (A)	>50	>50

Fom^R^26 (A)	>50	>50

Fom^R^1 (B)	25	2

Fom^R^9 (B)	50	5

Fom^R^10 (B)	50	5

Fom^R^11 (B)	50	5

Fom^R^12 (B)	>50	5

Fom^R^13 (B)	25	2

Fom^R^15 (B)	50	2

Fom^R^18 (B)	10	1

Fom^R^19 (B)	>50	2

Fom^R^23 (B)	50	2

WT (CHO)	1	1

Toy^R^-4 (CHO)	>100	>100

DrToy^R^-18 (CHO)	>100	>50

Fom^R^-4 (CHO)	1.5	>70

The degree of resistance of various cell lines towards formycin A and tubercidin was determined as described in Materials and Methods. Assuming the D_10 _values of these drugs for WT V79 cells as 1, the relative resistance of the mutant cell lines was calculated. (A) and (B) refer to the two class of mutants of V79 cells.

**Figure 3:**
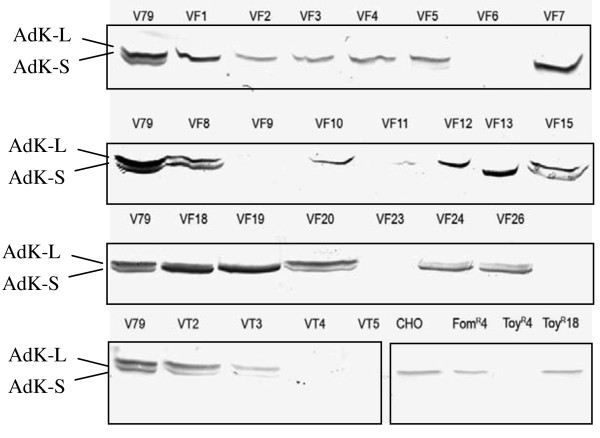
**Western blots showing the relative expression of the AdK-L and AdK-S short isoforms in various mutant cell lines**. Equivalent amount of cell extract (40 μg) from each of the cell lines was applied on the gels prior to electrophoresis and blotting. The WT refers to the parental V79 cells. VF and VT refer to various mutants selected using FoA and tubercidin, respectively. The results for the CHO cells and some of its mutant are present in the bottom right hand panel. Similar results for different mutants were obtained in at least two-independent experiments.

To determine if these mutants were affected in the expression of the AdK isoforms, Western blot analysis was performed (Figure 3). Two closely migrating protein bands with molecular masses of ~ 40-42 kDa corresponding to the two AdK isoforms were detected in the WT V79 cells and a number of mutants (VF8, VF15, VF18, VF19, VF20, VF24, VF26 and VT2 and VT3). However, large differences were observed in various mutants with regard to expression level of the two isoforms. VF13 mutant expresses only the AdK-S isoform, whereas in VF1-4 and VF10-12 mutants only the AdK-L isoform was found. In contrast to these mutants, several other mutants such as VT4, VT5, VF6, VF9 and VF23 showed no expression of either of the two AdK isoforms. Some of the other mutants including VF18, VF19, VF24 and VF26, also showed higher expression of the AdK-S isoform. Western blot analysis was also performed on some of the mutants of CHO cells that are studied in this work (Figure 3, bottom right hand panel). In the three mutants isolated from the CHO cells, which express only the AdK-L isoform, no cross-reactive protein was observed in the Toy^R^-4 mutant, whereas the DrToy^R^-18 and Fom^R^-4 mutants showed similar expression of the AdK-L isoform as the WT cells.

**Figure 4:**
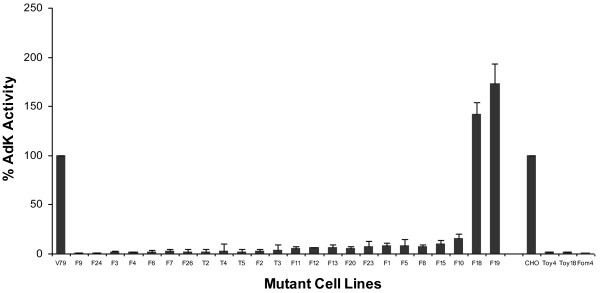
**AdK activity in the parental and mutant cell lines**. The protein concentration in all cell extracts was adjusted to be the same (1 mg/ml). Assuming the AdK activity in the parental V79 cells to be 100, the relative amount (%) of AdK activity in various mutants was determined. The results are average of at least 2 independent measurements.

The most common mechanism for resistance to Ado analogs involves deficiency or loss of AdK activity. Hence, AdK activity in the cell extracts of various mutants was measured and normalized with respect to both protein and AdK activity in the WT V79 cells. A summary of these results is presented in Figure 4. The cell extracts from twelve mutants (VT2-5 and VF2-4, VF6, VF7, VF9, VF24 and VF26) contain negligible (0.5-3%) AdK activity. The cell extracts from eleven other mutants (VF1, VF5, VF8, VF10-13, VF15, VF20 and VF23) contain low (6-15%), but significant level of AdK activity (Figure 4). In contrast to these mutants that contain either no or very low level of AdK activity, two other mutants VF18 and VF19 contain significantly higher amounts of AdK activity than in the WT V79 cells. The AdK activity was also examined in the three AdK mutants of CHO cells. All three of these mutants i.e. Toy^R^-4, DrToy^R^-18 and Fom^R^-4 contain only background activity (i.e. ≈ 0.5 -1% of the WT level). Of these mutants, the Toy^R^-4 contains a large deletion in the AdK gene. Hence, this level of background activity seen in the cell extracts of many mutants (Figure 4) is not due to AdK but it is possibly due to activities of other enzymes in cell extracts [24,29,32].

### Molecular Characterization of the Mutants affected in AdK

The mutants of CHO cells lacking in AdK activity that have been previously studied contained large deletions in the AdK gene [32] and they provided no useful information regarding structure-activity aspects of AdK. With the availability of a good antibody to AdK, the mutants lacking AdK antibody cross-reactive bands can now be readily identified and excluded from further analyses. These studies have revealed that the mutants DrToy^R^-18 and Fom^R^-4 isolated in our earlier work both contained normal amounts of the AdK antibody cross-reactive bands (see Figure 3). Hence, these mutants and two other mutants VF18 and VF19 obtained in this work were further characterized.

**Figure 5:**
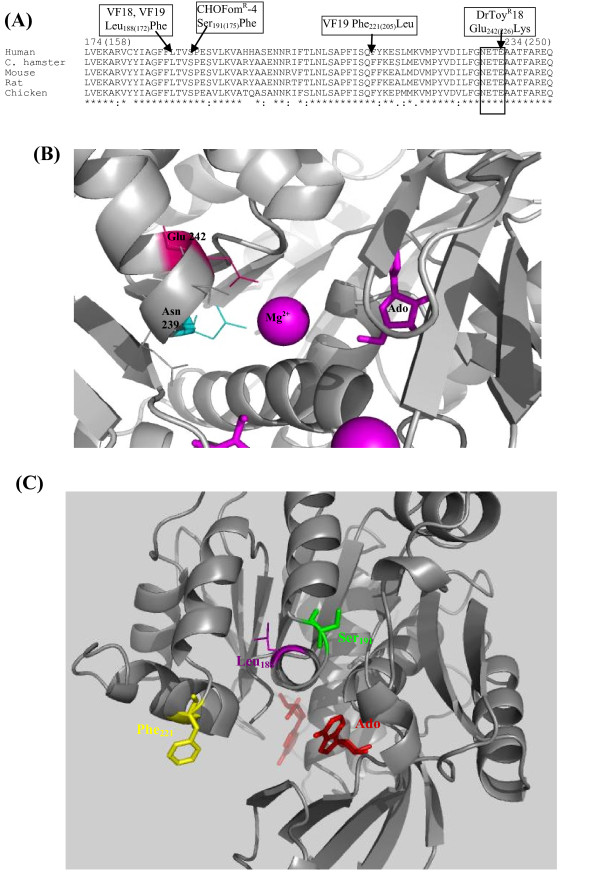
**Molecular and Structural alterations in a number of mutants affected in AdK.** (A) Partial sequence alignment of AdK from a number of species showing the conserved residues that are altered in the Fom^R^-4, VF18, VF19 and DrToy^R^-18 mutants. The NxxE motif in the sequence alignment is boxed. Of the two numbers shown for various amino acids that are altered in these mutants, the first corresponds to the position of the amino acids in the AdK-L isoform for Chinese hamster cells, whereas the latter numbers (in parenthesis) refer to their positions in the human AdK-S sequence, whose structure is shown below. (B) A close up view of human AdK structure [43] showing the position of the Glu_242(226) _residue that is altered in the DrToy^R^-18 mutant. Magnesium is shown as purple spheres and the conserved Asn_239(223) _and Glu_242(226) _residues of the NxxE motif are shown in magenta and blue colors, respectively. The proximity of the NxxE motif to Mg^2+ ^and the substrate adenosine is shown. (C) A close up view of the human AdK-S structure showing the locations of various amino acids that are altered in the Fom^R^-4, VF18 and VF19 mutants. The substrate Ado is shown in red.

Of these mutants, the mutant Fom^R^-4 is of particular interest. This mutant is highly resistant to FoA and other C-Ado analogs, but shows very little or no resistance to various N-Ado analogs including toyocamycin and tubercidin [31,34,41]. Interestingly, although this mutant was able to phosphorylate adenosine and various N-Ado analogs *in vivo*, the cell extracts from this mutant showed no AdK activity and all efforts to detect/recover AdK activity in its cell extracts have been unsuccessful [34]. Another novel aspect of this mutant is that its drug resistance phenotype expresses dominantly in cell hybrids formed with the WT CHO cells [34], which is in contrast to the recessive behaviour of all other AdK^- ^mutants that have been studied [29,41,42]. Hence, it was of much interest to determine the nature of the molecular alteration in this mutant. To characterize the molecular alterations in this and other mutants, full-length AdK sequences from the mutant cDNA were PCR amplified and sequenced. Of the four independent clones from the Fom^R^-4 mutant that were sequenced, all were found to contain a single C → T mutation that changed a conserved Ser residue at position 191 into phenylalanine. The changed amino acid corresponds to position 175 in the human AdK structure (Figure 5A) [43,44]. To confirm the significance of this molecular alteration for the biochemical and drug-resistance phenotype of the Fom^R^-4 mutant, the mutation causing S_191 _→ F alteration was introduced by site-directed mutagenesis into the WT CH (or human) AdK in the mammalian expression vector pcDNA3.1. The WT CHO cells and an AdK^- ^mutant of CHO cell (Toy^R^-4) were transformed with this plasmid DNA and stable transformants were selected in presence of the neomycin analog G418. The degree of resistance of these transformants as well as the WT CHO, Fom^R^-4 and Toy^R^-4 cell lines towards tubercidin and FoA was determined.

**Figure 6:**
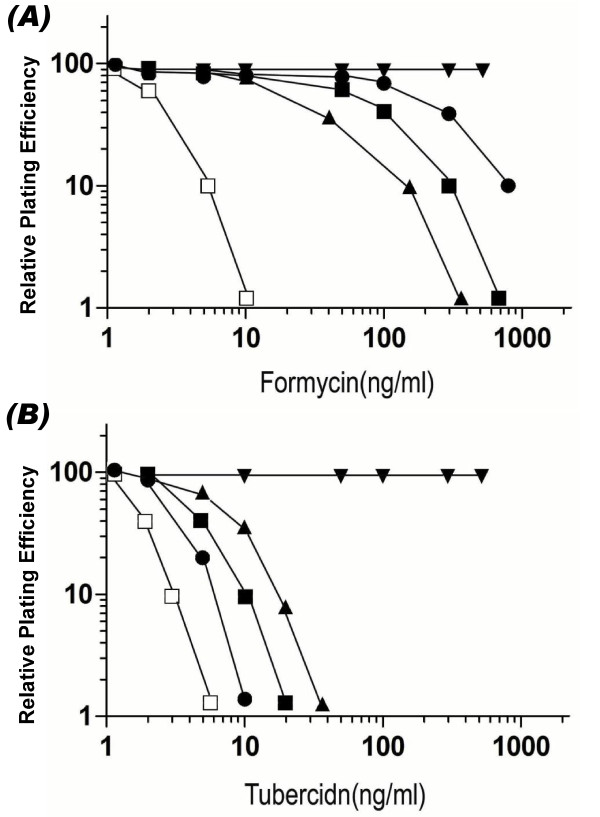
**Dose response curves for (A) Formycin A and (B) tubercidin for the WT CHO (□), Fom**^**R**^**-4 (●) and Toy**^**R**^**-4 (▼) cell lines and for one transformant clone selected from WT (■) and Toy**^**R**^**-4 (▲) cell lines after transfection with the expression plasmid containing AdK(Ser**_**191**_**Phe) mutation**. Similar results were obtained in at least 2 independent experiments.

The results of these experiments are shown in Figure 6. As expected, the WT CHO cells were sensitive to both tubercidin and FoA, whereas the Toy^R^-4 mutant containing a deletion in the AdK gene was highly resistant to both of them. Further, as reported in our earlier work [34], the Fom^R^-4 mutant was highly resistant to FoA but it displayed negligible resistance to tubercidin. The transformants obtained upon transfection of WT cells with the AdK(S_191_F) plasmid were interesting since they also exhibited resistant to FoA but were sensitive to tubercidin. Because WT CHO cells contained normal AdK, the FoA resistance of these transformants indicated that the mutant AdK(S_191_F) was able to confer resistance despite the presence of wild-type AdK. The results obtained upon transformation of the Toy^R^-4 mutant with the AdK(S_191_F) plasmid were equally interesting. Although the Toy^R^-4 mutant is highly resistant to both tubercidin and FoA, the transformants obtained in this case were found to be only marginally resistant to tubercidin and they displayed similar level of resistance to FoA as the Fom^R^-4 mutant. These results indicate that the expression of the AdK(S_191_F) plasmid has made the Toy^R^-4 cells sensitive towards tubercidin, while maintaining resistance towards formycin A. These results provided strong evidence that the S_191_F mutation in the AdK was behaving dominantly and it was responsible for the novel genetic and biochemical properties of the Fom^R^-4 mutant.

The DrToy^R^-18 mutant is highly resistant to both N- and C- adenosine analogs. The sequencing of AdK cDNA from this mutant revealed that it contained a single point mutation (G → A substitution), which changes a conserved Glu_242 _residue in the NxxE motif to Lys (Figure 5A). Earlier studies indicate that the conserved Asn and Glu residues in the NxxE motif (boxed in Figure 5A) are involved in the binding of activating phosphate ion, indicating that this motif is essential for AdK function [1,45]. We have also introduced the E_242_K mutation in CHO AdK cDNA in pET-15b expression vector [45]. Upon expression, the resulting protein showed no AdK activity (results not shown) confirming that this mutation leads to the inactivation of the enzyme and is responsible for the drug resistance phenotype of the DrToy^R^18 mutant. These results provide strong evidence that the NxxE motif is essential for AdK function *in vivo*.

The AdK cDNA was also PCR sequenced from the VF18 and VF19 mutants. For VF18 mutant, of the four clones sequenced, 2 contained a single point mutation changing the Leu_188 _into Phe. The remaining two clones showed no change in the AdK sequence. Similarly, of the 4 clones that were sequenced from the VF19 mutant, 2 contained the same mutation (Leu_188_Phe) as observed in the VF18 mutant. However, these clones in addition also contained a second mutation changing Phe_221 _into Leu. The mutant VF19 displays a higher degree of resistance to FoA in comparison to the VF18. Thus, it is possible that these two mutations synergistically reduce the binding of FoA to AdK. The locations of various amino acids that are altered in these mutants i.e. Ser_191_Phe, Leu_188_Phe, Phe_221_Leu and Glu_242_Lys in the human AdK structure are shown in Figure 5 B and 5C.

## Discussion and Conclusion

This paper presents information regarding several novel characteristics of AdK from mammalian cell lines. We show that the two isoforms of AdK differ from each other only in their first exons. Because each of these isoforms contains its own promoter, the transcription of these two isoforms should be regulated independently at the genetic level. The expression of the two isoforms also differs greatly in various rat tissues. Whereas in liver, kidney, lung and pancreas both isoforms were expressed at comparable levels, in other tissues such as heart, thymus, skeletal muscle and brain, either the AdK-L or AdK-S isoform is predominantly expressed. Large differences in the expression of these two isoforms have also been observed in earlier studies [12,22,23]. Importantly, our studies also showed that the expression of the two isoforms also differed greatly in established cell lines. Whereas the CHO and Hela cells expressed only the AdK-L isoform, in two other cell lines V79 and GM7S from the same species, both AdK isoforms were expressed in comparable amounts. It is of interest that the V79 and GM7 cell lines, which are derived from lung, expresses both isoforms. Therefore, it is possible that the expression of these isoforms in cultured cells reflect their expression profiles in corresponding tissues.

However, the main focus of this work was on isolation and characterization of mutant CH cell lines that are affected in AdK. From CH V79 cell line expressing both AdK isoforms, 24 mutants resistant to FoA and tubercidin were isolated. About half of these mutants were highly resistant to both tubercidin and FoA, whereas the remaining, although they were highly resistant to FoA, exhibited only marginal resistance to tubercidin. Biochemical studies have revealed that all of these mutants except VF18 and VF19 contained either no or very low level of AdK activity. This accounts for their resistance to the Ado analogs, which are converted into their toxic forms by AdK. The expression of the two AdK isoforms in different mutants also exhibited important differences. Whereas the mutants VF6, VF9, VT4 and VT5 containing only background AdK activity lacked both isoforms, many other mutants with similar level of AdK activity expressed either the AdK-S isoform viz. VF13, VF24 and VF26 or the AdK-L isoform viz. the mutants VF1-7 and VF12. Surprisingly, the mutants VF8, VF15, VF20 and VT2 that show negligible or greatly reduced level of AdK activity expressed both AdK isoforms at a level comparable to the WT V79 cells. Although, the molecular alterations in most of these mutants remain to be identified, it is quite likely that in mutants that do not express either of these isoforms, mutations or deletions affecting one or more of the common AdK exons (exons 2-11) have occurred. These mutants could be similar to the mutants CHO cells that have been previously characterized, which contained large deletions leading to loss of several introns and exons [32,36]. In contrast to these mutants, the mutants where expression of either the AdK-L or AdK-S isoform is preferentially affected are most likely to contain mutations in the promoter regions for these isoforms. Thus, further molecular characterization of these mutants, such as the methylation status, should provide useful insights concerning the functional significance of the two isoforms and how their expression may be regulated in mammalian cells.

The molecular alterations in a number of mutants resistant to Ado analogs were also identified in the present studies. One of these mutants, DrToy^R^-18 was specifically altered in the conserved NxxE motif, which has been indicated to be important in the binding of activating phosphate ion to AdK as well as other PfkB family of proteins [1,4,45,46]. The complete loss of AdK activity in this mutant, both *in vivo *and *in vitro*, now provides direct evidence that this motif, which is in close proximity to the binding sites for Mg^2+ ^ion as well the substrate adenosine (Figure 5B), is essential for AdK function *in vivo*.

In this work, we have also identified the molecular alteration in the Fom^R^-4 mutant, whose genetic and biochemical characteristics have remained puzzling for >25 years. We show that this mutant contains a single base substitution mutation that changes a conserved Ser_191 _into Phe. This mutation when introduced into either CH or human AdK (results not shown) was sufficient to confer a similar genetic and biochemical phenotype as observed for the Fom^R^-4 mutant. The Ser_191_Phe mutation is present in the AdK structure [43] on the periphery of the protein near the entrance of the substrate-binding pocket (Figure 5C). The C- Ado analogs, to whom the Fom^R^-4 mutant exhibits selective resistance exist predominantly in *Syn *conformation in contrast to the *Anti*-conformation for Ado and various N-nucleoside analogs [31,35]. Hence, it is likely that this molecular alteration selectively prevents the binding of FoA and other C-nucleosides to the mutant enzyme.

However, there are two other puzzling aspects of the Fom^R^-4 mutant that remain to be addressed. First, the cell extracts from this mutant show no AdK activity despite its containing AdK activity *in vivo*. Second, the drug-resistance phenotype of this mutant is dominant in cell hybrids formed with WT cells [34,41]. The first observation suggests that the reaction product of AdK i.e. AMP is not released from the mutant enzyme under *in vitro *conditions, but in the cellular milieu it is likely directly transferred to the next enzyme AMP-kinase (AMPK) in the pathway. The AMPK, which carries out the reaction AMP + ATP ↔ 2 ADP, plays a key role in maintaining the equilibrium concentrations of all three adenine nucleotides [47]. The subsequent phosphorylation of ADP into ATP is carried out by the enzyme nucleoside diphosphate kinase [48]. Because the toxicity of Ado analogs (e.g. FoA) requires their phosphorylation into higher phosphorylated forms (e.g. di- and tri-phosphates), which interfere with different metabolic pathways [31,35,49], if the mutation in AdK prevents the conversion of FoA or FoA-PO_4 _into higher phosphorylated forms then their toxicity will be averted.

**Figure 7:**
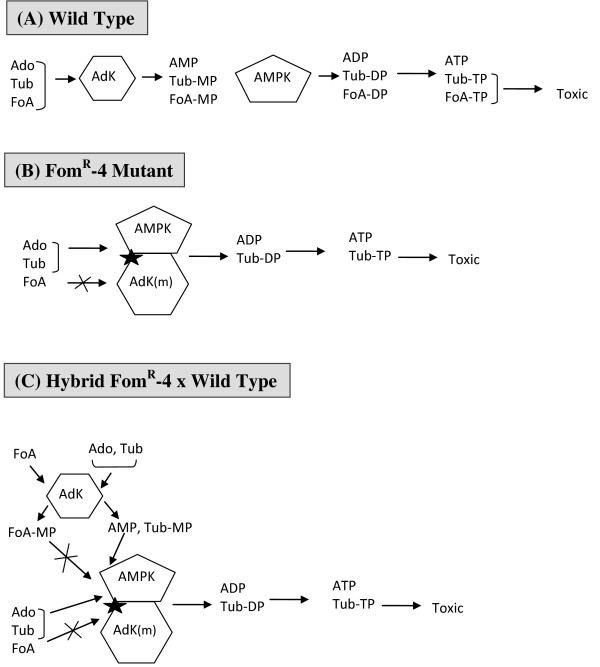
**A model to account for the lack of AdK activity in the cell extracts of the Fom**^**R**^**-4 mutant and dominant expression of its drug-resistance phenotype**. (A) In the WT cells, AdK converts Ado and various Ado-analogs (e.g. FoA, Tub) into their corresponding monophosphates; subsequently AMP-kinase (AMPK) and other enzymes convert them into di- and tri-phosphates. (B) and (C), In the Fom^R^-4 mutant or the cell hybrids formed between Fom^R^-4 and the WT cells, the Ser_191_Phe mutation in AdK (indicated by ✭) leads to its complex formation with AMPK. This mutation is also postulated to specifically prevent the binding of FoA and FoA-MP to the AdK-AMPK complex. As a result of this complex formation, AMP (or Tub-MP) formed by AdK is not released but directly transferred to the AMPK for conversion into ADP. These account for the unusual properties of the Fom^R^-4 mutant.

In view of these consideration, to account for the lack of AdK activity in the Fom^R^-4 extracts and the dominant expression of this mutation, we postulate that the mutant AdK and the enzyme AMPK exist as a complex in cells such that the AMP formed from AdK reaction is not released but directly transferred to AMPK for conversion into ADP (Figure 7). Further, although this complex binds adenosine, N-Ado analogs and the monophosphates of N-adenosine analogs (i.e. Toy-MP or Tub-MP) normally, it is unable to bind FoA or FoA-MP as a result of the Ser_191_Phe mutation. The formation of a complex between these two enzymes will explain both the lack of AdK activity in the mutant cell extracts (as the product AMP is not released from the mutant enzyme) and also the dominant expression of FoA resistance in this mutant. In cell hybrids formed between WT and Fom^R^-4, although the WT AdK can phosphorylate FoA, the FoA-MP formed, due to its non-recognition by the AdK-AMPK complex, is not further metabolized to higher phosphorylated forms to cause toxic effect. Thus, these cells display resistance to FoA. It should be noted that the Ser_191_Phe mutation in the Fom^R^-4 mutant is present on the surface of AdK (Figure 5C) and it replaces a hydrophilic amino acid with a hydrophobic residue thus creating a surface hydrophobic patch (denoted by ⋆ in Figure 7) that could be important in its complex formation with the AMPK. Although, this model is speculative, based upon our results, it is the only model that can account for the different characteristics of the Fom^R^-4 mutant, which has remained an enigma for >25 years. However, this model makes a number of predictions, which will be experimentally tested, providing further insights into the cellular function of this enzyme.

Lastly, we have also identified the molecular alterations in two of the V79 cell mutants, V18 and VF19, which similar to the Fom^R^-4 mutant also exhibit preferential resistance to the C-Ado analogs. The observed molecular alterations in these two mutants are located on the same face of AdK as the mutation in the Fom^R^-4 mutant (Figure 5C). Thus, it is likely that the genetic changes in these mutants, Leu_188_Phe and Phe_221_Leu, also selectively affect the binding of FoA to the mutant AdK. However, it is of interest that of the four clones that were sequenced for these mutants, only two contained mutational alterations, whereas the other two showed no change. This is in contrast to the Fom^R^-4 mutant, where all four sequenced clones contained the Ser_191_Phe mutation. Earlier work on CHO and V79 cells has provided evidence that the CHO cell line is functional hemizygous for many genetic loci including the AdK gene, whereas in V79 cells two functional copies of these genes were inferred [29,50]. In this context, our observations that in the Fom^R^-4 cells only the mutated form of AdK was found, whereas in mutant V79 cells both WT and the mutated forms of AdK were present support to this inference. The fact that the VF18 and VF19 mutants are resistant to FoA, despite their containing normal levels of AdK activity, indicates that the mutations in them are also expressing codominantly.

The mutants VF18 and VF19 are also of much interest, because in comparison to all other AdK mutants, they are the only mutants exhibiting enhanced AdK activity relative to the parental V79 cells. These mutants also show higher expression of the AdK-S isoform in comparison to the AdK-L isoform. These observations indicate that these mutants, in addition to the molecular changes that we have identified in this work, also contain additional genetic changes affecting the expression of the two isoforms. To understand the functional significance of different molecular alterations in these mutants, it is of much interest to further characterize AdK from these mutants at genetic, molecular and biochemical levels.

## Methods

### Cell Culture and Cell Lines

The origins of various CH cell lines CHO, V79 and GM7S used in this work have been described in earlier work [29,39]. The Toy^R^-4, DrToy^R^-18 and Fom^R^-4 mutants of CHO cells were also isolated and partially characterized in earlier work [24,32,34]. Of these Toy^R^-4 and DrToy^R^-18 mutants are highly resistant to both N- and C-Ado analogs. The mutant DrToy^R^-18 was obtained from the Dr-31 cell line, which is a different clone of the original CHO cell line [29]. The Toy^R^-4 mutant has previously been shown to contain a large deletion in the AdK gene [24,29,32], whereas the genetic lesion in DrToy^R^-18 has not yet been identified. The Fom^R^-4 mutant was selected using FoA and its various characteristics are described in earlier work [34,41]. The cells were grown in monolayer culture at 37°C in alpha medium supplemented with 5% fetal bovine serum in 95% air - 5% CO_2 _atmosphere. For selection of mutants, V79 cells were treated with 300 μg/ml of the mutagen ethyl methanesulfonate for 18 hrs and then grown for 5 days [29]. The mutants were selected by plating 5 × 10^5 ^cells/dish in multiple dishes in medium containing either 200-500 ng/ml FoA (+ 10 μg/ml of the adenosine deaminase inhibitor erythro-9-(2-hydroxy-3-nonyl)adenine) or 80 ng/ml tubercidin. The mutant colonies were expanded and maintained by growth in non-selective medium. The mutants were named based on the selective drug. Thus, V79Fom^R ^and V79Tub^R ^(annotated as VF and VT) denote mutants isolated using FoA and tubercidin. The degree of resistance of the cell lines towards Ado analogs was determined by plating 200 and 500 cells in medium containing different concentrations of the analogs as in earlier work [29,34]. After 7 days, the colonies were fixed, stained with 0.5% methylene blue and their numbers were counted. Assuming the number of colonies formed in the absence of any drug to be 100%, the relative plating efficiencies of cell lines in presence of different drug concentrations were determined. The D_10 _value represents the drug concentration that reduces plating efficiency of a cell line to 10% of that observed in the absence of any drug [40]. The sources of various Ado analogs and other chemicals have been described in earlier work [29,34].

### Adenosine kinase activity assay, Western blotting, RT-PCR and Sequencing

AdK activity was measured as described previously using a radioactive assay involving conversion of [^3^H]-adenosine to [^3^H]-AMP [29,45]. [2,8-^3^H]-Adenosine (40 Ci/mmol) was purchased from American Radiolabeled Chemicals Inc. For Western blot analysis, 40 μg of cell extracts from different cell lines were electrophoresed on 12% sodium dodecyl sulfate polyacrylamide gels (SDS-PAGE). After transfer to nitrocellulose and blocking, the blot was reacted with 1:1000 dilution of a rabbit polyclonal antibody to human recombinant AdK raised in our lab [27]. After washing the blot was reacted with 1:2000 dilution of anti-rabbit lgG conjugated to horseradish peroxidase and then developed using 4-Chloro-1-naphthol and H_2_O_2_. All of the experiments were repeated at least twice with very similar results. The quantification of the results for the rat tissue experiments (three independent experiments) was carried out using the NIH ImageJ software and the average intensity ± SD was calculated. For RT-PCR, total RNA was isolated from 1-2 confluent dishes using TRIzol (Invitrogen) as per the manufacturer's protocol. cDNA was generated using RevertAid™ H Minus First Strand cDNA Synthesis Kits (Fermentas) with oligo(dT)18 primers. Full length AdK sequence was amplified from the cDNA using the forward primer 5'-ATGGCAGCTGCTGAGGAGC-3' and reverse primer 5'-TCAGTGGAAGTCTGGCTTCTC-3' based on CH AdK sequence [26]. The amplified fragments were cloned and sequenced using the M13 forward and reverse primers.

### Mammalian Cell Transfection and In vitro Mutagenesis

The full-length AdK cDNA (long isoform) from CH was cloned in the mammalian cell expression vector pcDNA3.1 [26]. The S_191_→F mutation in the CH (or human) cDNA was made using the 'Quickchange' site-directed mutagenesis kit (Stratagene) as described in earlier work [27,45]. The transfection of the wild-type (WT) or the mutant Toy^R^-4 CHO cells with these plasmid DNAs was carried out using the Lipofectamin-2000 reagent (Invitrogen) as described in earlier work [27]. Stable transfectants expressing these genes were obtained by growing the cells in presence of G-418 (650 μg/ml) for more than 1 month. The degree of resistance of these transformants for tubercidin and FoA was determined as described above.

## Authors' contributions

XAC and TA carried out selection of various mutants of V79 cells and cross-resistance studies, enzyme activity assay and immunoblot analyses on them. XAC and BS were responsible for examining the tissue distribution of AdK and for molecular characterization of the mutants. BS was responsible for identifying the promoter region for the AdK-S isoform, for creation of site-directed mutants and for transformation studies on them. RSG was responsible for conceiving and directing this study and for writing the manuscript, which has been seen and approved by all authors.
